# Poor Sleep Quality Associated With Enlarged Perivascular Spaces in Patients With Lacunar Stroke

**DOI:** 10.3389/fneur.2021.809217

**Published:** 2022-01-28

**Authors:** Shuna Yang, Jiangmei Yin, Wei Qin, Lei Yang, Wenli Hu

**Affiliations:** ^1^Department of Neurology, Beijing Chaoyang Hospital, Capital Medical University, Beijing, China; ^2^Department of Neurology, Beijing Pinggu District Hospital, Beijing, China

**Keywords:** enlarged perivascular spaces, Virchow-Robin spaces, cerebral small vessel diseases, Pittsburgh Sleep Quality Index, sleep quality

## Abstract

**Background and Objective:**

Enlarged perivascular spaces (EPVSs) are considered as an MRI marker of cerebral small vessel diseases and were reported to be associated with brain waste clearance dysfunction. A previous study found that interstitial fluid clearance in the mouse brain occurred mainly during sleep. However, the relationship between sleep quality and EPVS in humans has not been well-understood. Thus, we aimed to investigate the relationship between sleep and EPVS in humans.

**Methods:**

This retrospective study was conducted in patients with lacunar stroke in the Neurology Department of Beijing Chaoyang Hospital. Patients with EPVS >10 on one side of the basal ganglia (BG) and white matter slice containing the maximum amount were defined as the BG-EPVS group and the white matter (WM)-EPVS group, respectively. Patients with EPVS <10 in the slice containing the maximum amount were defined as the control group. Sleep quality was evaluated by the Pittsburgh Sleep Quality Index (PSQI) including seven components, where a score of 6 or higher indicated poor sleep quality. Spearman's correlation analysis and the binary logistic regression analysis were performed to analyze the relationship between poor sleep quality and BG-EPVS and WM-EPVS, respectively.

**Results:**

A total of 398 patients were enrolled in this study, including 114 patients in the BG-EPVS group and 85 patients in the WM-EPVS group. The proportion of poor sleep quality in the BG-EPVS group was higher than that in the control group (58.8 vs. 32.5%, *p* < 0.001). The score of PSQI, subjective sleep quality, sleep latency, sleep duration, and sleep efficiency were higher in the BG-EPVS group than that in the control group (*p* < 0.05). The proportion of poor sleep quality was also higher in the WM-EPVS group than that in the control group (50.6 vs. 35.3%, *p* = 0.031). The score of sleep duration and sleep disturbances was higher in the WM-EPVS group than that in the control group. Spearman's correlation analysis showed that poor sleep quality was positively associated with BG-EPVS (ρ = 0.264, *p* < 0.001) and WM-EPVS (ρ = 0.154, *p* = 0.044). The binary logistic regression analysis showed that poor sleep quality, longer sleep latency, and less sleep duration were independently related to BG-EPVS and poor sleep quality, less sleep duration, and more serious sleep disturbances were independently related to WM-EPVS after adjusting for confounders (*P* < 0.05).

**Conclusion:**

Poor sleep quality was independently associated with EPVS in BG and WM.

## Introduction

Perivascular spaces, or Virchow–Robin spaces, are perivascular compartments surrounding the small cerebral penetrating vessels, serving as a protolymphatic system and playing an important role in interstitial fluid and solute clearance in the brain. They will dilate with the accumulation of interstitial fluids (1). Enlarged perivascular spaces (EPVSs), visible on MRI, appear as punctate or linear signal intensities similar to cerebrospinal fluid (CSF) on all the MRI sequences in white matter (WM-EPVS), basal ganglia (BG-EPVS), hippocampus, and brainstem (2, 3). There are some differences in the anatomical structure, mechanisms, and risk factors of BG-EPVS and WM-EPVS (4). Now, EPVS are considered as an MRI marker of cerebral small vessel diseases and are associated with age, hypertension, white matter hyperintensities (WMH), brain atrophy, and lacunes (2, 5). Some studies found that EPVSs were associated with impaired cognitive function (6), incident dementia (7), and Parkinsonism syndrome (8, 9). Therefore, it is very important to understand the pathogenesis and risk factors for EPVS.

Xie et al. (10) found that interstitial fluid clearance in the mouse brain occurred mainly during sleep, which suggested a homeostatic function of sleep through removing waste generated from neuronal metabolism. It is reasonable that poor sleep quality in humans may disrupt the removal of neurotoxins, interrupt the drainage of interstitial fluid, and possibly result in dilation of the perivascular spaces. In addition, some studies have demonstrated that poor sleep quality was associated with brain atrophy and WMH which share some risk factors with EPVS (11, 12). However, the relationship between EPVS and sleep in humans is scarcely explored. In this study, we aimed to explore whether sleep quality is associated with BG-EPVS and WM-EPVS.

## Materials and Methods

**Figure 1:**
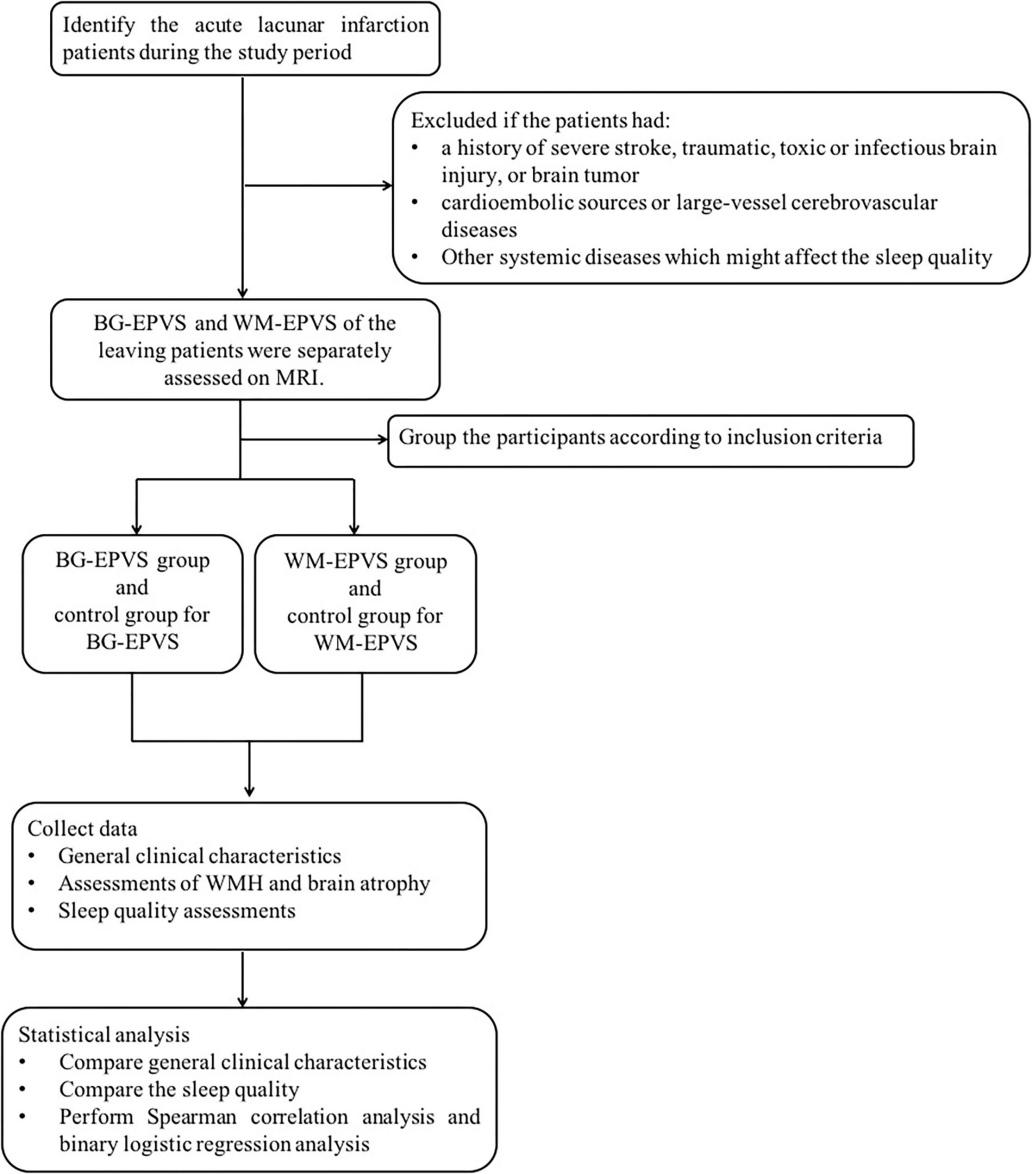
Research flow chart. BG-EPVS, enlarged perivascular spaces in basal ganglia; WM-EPVS, enlarged perivascular spaces in white matter; WMH, white matter hyperintensities.

Figure 1 was the research flowchart and presented the research process.

### Study Subjects

This retrospective study was conducted as a case-control study in patients with lacunar stroke. We identified all patients with acute lacunar stroke admitted to the Neurology Department of Beijing Chaoyang Hospital affiliated to Capital Medical University from April 2015 to May 2017. Lacunar stroke was confirmed by MRI and defined as a lesion of increased signal on axial diffusion-weighted imaging (DWI) ≤ 20 mm in the distribution of a small penetrating artery. Patients with lacunar stroke were excluded if: (1) they had a history of severe ischemic or hemorrhagic stroke (the largest diameter of infarct size >20 mm) on DWI and fluid-attenuated inversion recovery (FLAIR), traumatic or toxic or infectious brain injury, and brain tumor because of affecting assessments on EPVS; (2) they had possible cardioembolic sources or large-vessel cerebrovascular diseases defined as internal carotid, middle cerebral, or basilar intracranial artery stenosis >50%; (3) they had other systemic diseases including recent myocardial infarction or angina pectoris disorders, heart failure, infections, nephrosis, pulmonary diseases, liver diseases, or tumor which might affect the sleep quality.

**Figure 2:**
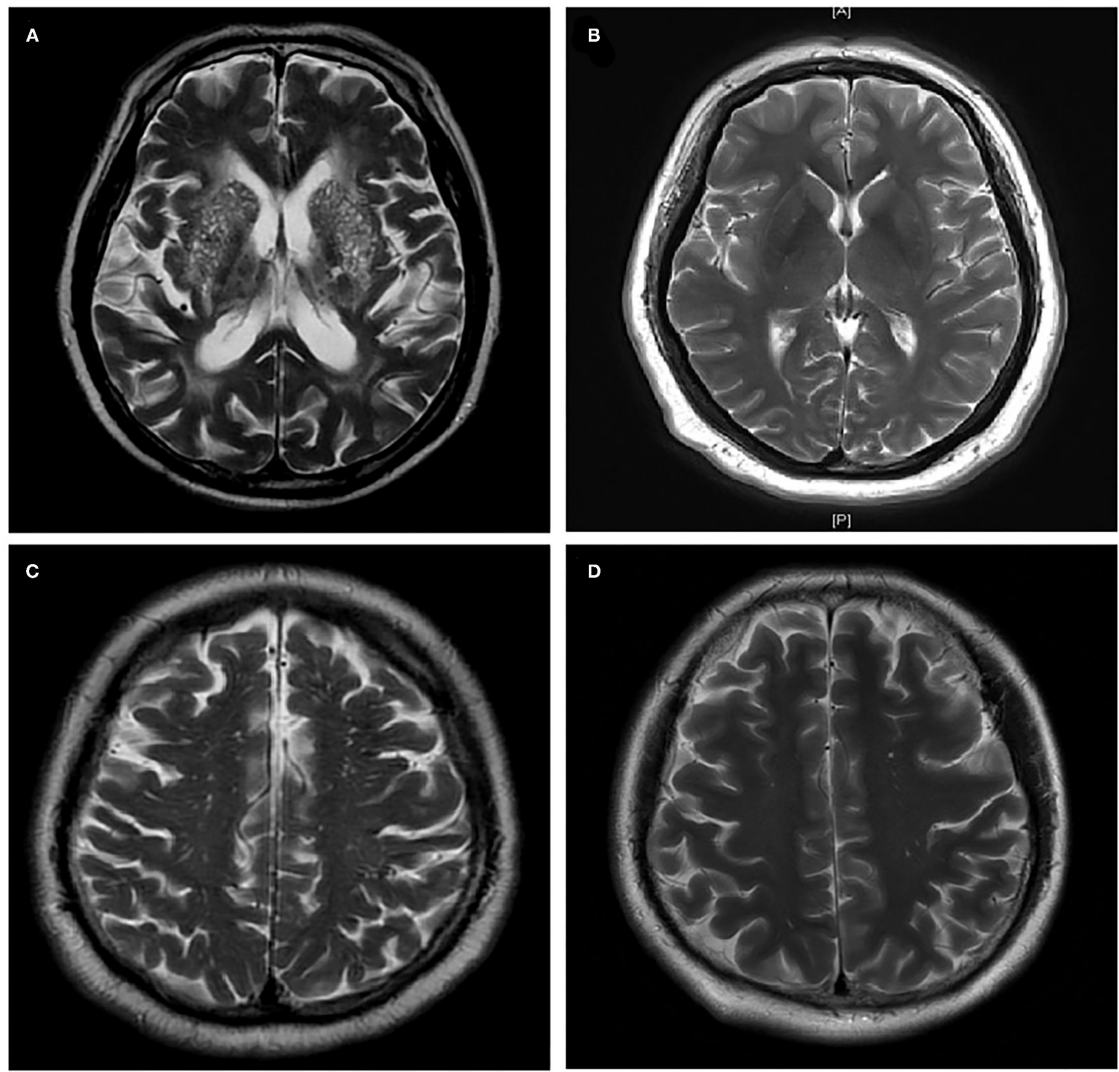
Enlarged perivascular spaces group and control group. **(A)** Enlarged perivascular spaces in basal ganglia (BG-EPVS) group (Patients with EPVS >10 on one side of the basal ganglia slice containing the maximum amount); **(B)** control group for BG-EPVS (Patients with EPVS <10 on one side of the basal ganglia slice containing the maximum amount); **(C)** enlarged perivascular spaces in white matter (WM-EPVS) group (Patients with EPVS >10 on one side of the white matter slice containing the maximum amount); **(D)** control group for WM-EPVS (Patients with EPVS <10 on one side of the white matter slice containing the maximum amount).

The BG-EPVS group, the WM-EPVS group, and the control group were selected from the leaving patients with lacunar stroke according to the following inclusion criteria. Inclusion criteria of the BG-EPVS group: (1) Patients with BG-EPVS >10 on one side of the basal ganglia slice containing the maximum amount (Figure 2A); (2) agreed to participate in this study; (3) finished the sleep assessments. Inclusion criteria of the WM-EPVS group: (1) Patients with WM-EPVS >10 on one side of the white matter slice containing the maximum amount (Figure 2C). The other inclusion criteria were the same as that of the BG-EPVS group. Inclusion criteria of the control group: (1) Patients with EPVS <10 on one side of the slice containing the maximum amount (Figures 2B,D). The other inclusion criteria were the same as that of the BG-EPVS group. A subset of controls was, respectively, matched to the BG-EPVS group and the WM-EPVS group by age (±2 years) and sex, with one control for each case. This was due to the fact that age and sex have been found to be associated with EPVS. The cutoff >10 EPVS was used as a high grade in our previous studies and showed an excellent intrarater Cohen *k* score (13, 14).

### Ethical Standard Statement

This study was approved by the Ethics Committee of Beijing Chaoyang Hospital Affiliated to Capital Medical University and was conducted in accordance with the Declaration of Helsinki. All the participants provided written informed consent.

### General Clinical Characteristics Assessments

Age, sex, body mass index (BMI), past medical history including history of hypertension, diabetes mellitus, current smoking, and current alcohol consumption were collected. All blood samples were collected in the morning after an overnight and sent to the clinical laboratory of our hospital for the measurement of serum indices. Laboratory tests included total cholesterol (TC), triglyceride, high-density lipoprotein (HDL), low-density lipoprotein (LDL), hemoglobin A1c (HbA1c), blood urea nitrogen (BUN), and creatinine.

### MRI Examinations and Assessments of EPVS, WMH, and Brain Atrophy

The neurological image examinations were performed in the Radiology Department of our hospital. MR imagines were acquired on a 3.0 T MR scanner (Siemens, Erlangen, Germany). MRI sequences included axial T1-weighted, axial T2-weighted, axial DWI, and coronal FLAIR.

Enlarged perivascular spaces were defined as CSF-like signal intensity lesions of round, ovoid, or linear shape of <3 mm and located in areas supplied by perforating arteries. We distinguished lacune from EPVS by their larger size (>3 mm), spheroid shape, and surrounding hyperintensities on FLAIR (2). BG-EPVS and WM-EPVS were separately assessed according to the number in the slice containing the maximum amount.

White matter hyperintensities were scored by the Fazekas scale. A detailed description of the assessment has been previously published (15). Periventricular and deep WMH were evaluated separately and totaled together as Fazekas scores. Brain atrophy was evaluated according to the visual rating scale of global cortical atrophy (GCA) (16). In mild brain atrophy (point 1) there is sulcal opening peripherally, moderate brain atrophy (point 2) is characterized by widening along the length of the sulci, and severe brain atrophy (point 3) is present when there is gyral thinning.

The intrarater agreement for the rating of EPVS, WMH, and brain atrophy was assessed on a random sample of 50 individuals with a month interval between the first and second readings. Assessments of EPVS, WMH, and brain atrophy were performed by two experienced neurologists blinded to clinical information to avoid bias. Random scans of 50 individuals were independently examined by the two experienced neurologists blinded to each other's readings. The *k* statistics of intrarater and interrater agreement was 0.80 or above, indicating good reliability. The disagreement was resolved by discussing it with other co-authors.

### Sleep Quality Assessment

Sleep quality was assessed by the Pittsburgh Sleep Quality Index (PSQI) (17), which is a self-rated questionnaire. It assesses sleep quality and disturbances in the previous month and consists of 19 individual items generate seven components scores: subjective sleep quality, sleep latency, sleep duration, sleep efficiency, sleep disturbances, use of sleeping medication, and daytime dysfunction due to sleepiness (maximum score is 21 points). The cutoff value for poor sleep quality is ≥6 points (18). The PSQI questionnaire was conducted seven days after the onset of lacunar stroke.

### Statistical Analysis

Continuous variables were summarized as mean values ± SD or median (interquartile range) according to whether their distribution conformed to a normal distribution. Categorical variables were presented as absolute numbers and percentages. Continuous variables with both normal distribution and homogeneity of variance were compared with Student's *t*-test, whereas were compared with the Wilcoxon rank-sum test. The chi-squared test was used for the comparison of categorical variables. Spearman correlation analysis was performed to observe the correlation between poor sleep quality and EPVS. Grouping was considered as the dependent variable and binary logistic regression analysis was performed to explore if poor sleep quality was independently related to BG-EPVS and WM-EPVS after adjusting for confounding factors. Analysis was performed with Statistical Package for Social Sciences (SPSS) (version 21.0), and statistical significance was accepted at *p* < 0.05.

## Results

### General Clinical Characteristics of Participants

During the study period, 482 patients with acute lacunar infarction were identified. However, 20 patients were excluded because of the history of severe ischemic or hemorrhagic stroke, 35 patients were excluded because of large-vessel cerebrovascular diseases, 5 were excluded because of a history of tumor, and 7 were excluded because of atrial fibrillation. Of the leaving 415 patients with lacunar infarction, 114 patients were enrolled into the BG-EPVS group and 85 patients were enrolled into the WM-EPVS group. The same number of age and sex-matched controls were also recruited in the same population. Finally, 398 patients were enrolled in the study. The mean age of the cohort was 68 ± 9.8 years and 221 (55.5%) of them were men. In total, 142 subjects were current smokers and 78 were current alcohol users. In total, 300 subjects had a history of hypertension, and 171 had diabetes. The general clinical characteristics of the BG-EPVS group, the WM-EPVS group, and the control group are given in Table 1.

**Table 1:** General clinical characteristics of participants.

**Characteristics**	**BG-EPVS group**	**control group for BG-EPVS**	***P***	**WM-EPVS group**	**control group for WM-EPVS**	***P***
n	114	114	–	85	85	–
Age^a^, years	71 ± 8.5	69 ± 9.4	0.174	68 ± 9.8	70 ± 9.3	0.295
Sex, male (%)	69 (60.5)	45 (39.5)	0.410	58 (68.2)	49 (57.6)	0.204
BMI^a^, kg/m^2^	24.9 ± 2.8	25.4 ± 3.0	0.168	25.4 ± 2.8	25.2 ± 3.2	0.759
Current smoking (%)	31 (26.3)	53 (46.5)	0.003	37 (43.5)	21 (24.7)	0.010
Current alcohol (%)	23 (20.2)	26 (22.8)	0.603	17 (20.0)	12 (14.1)	0.308
Hypertension (%)	90 (78.9)	79 (69.3)	0.096	70 (82.4)	61 (71.8)	0.101
Diabetes (%)	45 (39.5)	54 (47.4)	0.229	36 (42.4)	36 (42.4)	1.000
TC^a^, ^b^, mmol/L	4.3 ± 1.04	4.5 ± 1.22	0.116	4.35 (3.64–5.11)	4.22 (3.58–5.08)	0.589
Triglyceride^b^, mmol/L	1.32 (1.01–1.86)	1.40 (1.05–2.06)	0.331	1.35 (1.03–2.05)	1.32 (0.95–1.84)	0.476
HDL^b^, mmol/L	1.13 (0.93–1.30)	1.10 (0.90–1.30)	0.604	1.10 (0.90–1.30)	1.20 (1.00–1.40)	0.136
LDL^a^, mmol/L	2.60 ± 0.83	2.70 ± 0.93	0.711	2.77 ± 0.86	2.56 ± 0.83	0.100
HbA1^b^, %	6.0 (5.6–6.8)	6.8 (5.8–8.0)	0.001	6.2 (5.6–7.6)	6.1 (5.7–7.2)	0.875
BUN^a^, ^b^, mmol/L	5.53 (4.50–6.85)	4.85 (3.84–6.24)	0.003	5.63 ± 1.98	5.72 ± 1.70	0.307
Creatinine^b^, umol/L	68.4 (56.8–81.7)	64.6 (54.5–79.7)	0.294	68.5 (56.2–84.4)	66.3 (54.7–77.9)	0.362
WMH^b^, point	5 (4–6)	2 (2–4)	<0.001	3 (2–6)	4 (2–6)	0.196
Brain atrophy^b^, point	1 (1–2)	1(0–1)	<0.001	1 (0–1)	1 (0–2)	0.186

*BMI, body mass index; TC, total cholesterol; HDL, high-density lipoprotein; LDL, low-density lipoprotein; AST, aspartate aminotransferase; ALT, alanine aminotransferase; HbA1c, hemoglobin A1c; BUN, blood urea nitrogen; WMH, white matter hyperintensities*.

a *Continuous variables with normal distribution were expressed as mean values ± standard deviation and were compared with Student's t-test*.

b *Continuous variables with non-normally distribution were expressed as median (interquartile range) and compared with Wilcoxon rank-sum test*.

There were no statistical significances in BMI, the proportion of current alcohol, hypertension, and diabetes between the BG-EPVS group and the control group. The proportion of current smoking in the BG-EPVS group was lower than that in the control group. The BG-EPVS group had higher level of blood urea nitrogen (median: 5.53 vs. 4.85 mmol/l, *p* = 0.003) and lower level of HbA1c (median: 6.0 vs. 6.8%, *p* = 0.001). Considering the imaging characteristics, the BG-EPVS group had more serious WMH [Fazekas scale: 5 (4–6) vs. 2 (2–4), *p* < 0.001] and brain atrophy [GCA scale: 1 (1–2) vs. 1 (0–1), *p* < 0.001].

The comparative results of general clinical characteristics between the WM-EPVS group and the control group were different from those between the BG-EPVS group and the control group. The proportion of current smoking in the WM-EPVS group was higher than that in the control group (43.5 vs. 24.7%, *p* = 0.010). There were no statistical significances in the proportion of current alcohol, hypertension, and diabetes, the levels of laboratory tests, and imaging characteristics between the WM-EPVS group and the control group.

### Association Between Sleep Quality and BG-EPVS

The PSQI score, proportion of poor sleep quality, and scores of sleep components including subjective sleep quality, sleep latency, sleep duration, sleep efficiency, sleep disturbances, use of sleeping medication, and daytime dysfunction due to sleepiness are given in Table 2.

**Table 2:** Sleep variables by Pittsburg Sleep Quality Index of the enlarged perivascular space (EPVS) group and the control group.

**Sleep variables**	**BG-EPVS group**	**control group for BG-EPVS**	***P***	**WM-EPVS group**	**control group for BG-EPVS**	***P***
Pittsburgh Sleep Quality Index, point	6 (4–10)	4 (3–7)	<0.001	6(3–8.5)	5(3–7.5)	0.165
Poor sleep quality (%)	58.8	32.5	<0.001	50.6	35.3	0.031
Subjective sleep quality, point	1 (1–2)	1 (0–1)	0.001	1(1–2)	1(1–2)	0.281
Sleep latency, point	1 (0–3)	1 (0–1)	0.004	1(0–2)	1(0–2)	0.935
Sleep duration, point	1 (0–2)	1 (0–1)	0.003	1(0–2)	1(0–1.5)	0.025
Sleep efficiency, point	1 (0–2)	0 (0–1)	0.021	1(0–2)	1(0–1)	0.211
Sleep disturbances, point	0 (0–1)	0 (0–1)	0.545	1(0–1)	0(0–1)	0.006
Use of sleeping medication, point	0 (0–0)	0 (0–0)	0.054	0(0–0)	0(0–0)	1.000
Daytime dysfunction, point	1 (1–2)	1 (0–2)	0.124	1(0–2)	1(0–2)	0.655

*The above non-normal distributed continuous variables were expressed as median (interquartile range) and compared with the Wilcoxon rank-sum test*.

The BG-EPVS group had the higher PSQI score and a higher proportion of poor sleep quality (PSQI score ≥6 points) than the control group [PSQI score: 6 (4–10) vs. 4 (3–7), *p* < 0.001, poor sleep quality: 58.8 vs. 32.5%, *p* < 0.001]. Spearman's correlation analysis showed that the PSQI score and poor sleep quality were positively associated with BG-EPVS (PSQI score: ρ = 0.248, *p* < 0.001, poor sleep quality: ρ = 0.264, *p* < 0.001). The results of the binary logistic regression analysis showed that poor sleep quality was independently related to BG-EPVS after adjusting for current smoking, level of HbA1c, blood urea nitrogen, WMH, and brain atrophy. The detailed analysis results are shown in Table 3.

**Table 3:** Results of the binary logistic regression analysis between sleep quality and enlarged perivascular spaces in basal ganglia (BG-EPVS).

**Sleep variables**	**B**	***P***	**Odds ratio**	**95% Confidence intervals**
Poor sleep quality (%)	0.754	0.022	2.125	1.113–4.058
Sleep latency	0.293	0.048	1.340	1.003–1.791
Sleep duration	0.360	0.024	1.434	1.050–1.958
Sleep efficiency	0.244	0.120	1.276	0.938–1.735

*Binary logistic regression analysis was performed adjusting for current smoking use, level of hemoglobin A1c and blood urea nitrogen, WMH, and brain atrophy*.

Furthermore, we analyze the relation between sleep components and BG-EPVS. The score of subjective sleep quality [1 (1–2) vs. 1 (0–1), *p* = 0.001], sleep latency [1 (0–3) vs. 1 (0–1), *p* = 0.004], sleep duration [1 (0–2) vs. 1 (0–1), *p* = 0.003], and sleep efficiency [1 (0–2) vs. 0 (0–1), *p* = 0.021] were higher in the BG-EPVS group than that in the control group, which mean that the BG-EPVS group had longer sleep latency, less sleep duration, and lower sleep efficiency. There was no statistical significance in the score of sleep disturbances, use of sleeping medication, and daytime dysfunction between the BG-EPVS group and the control group. The results of the binary logistic regression analysis showed that longer sleep latency and less sleep duration were independently related to BG-EPVS after adjusting for current smoking, level of HbA1c, blood urea nitrogen, WMH, and brain atrophy. The detailed analysis results are shown in Table 3.

### Association Between Sleep Quality and WM-EPVS

The PSQI score, proportion of poor sleep quality, and sleep components score of the WM-EPVS group and the control group for WM-EPVS are shown in Table 2.

Although there was no statistical difference in PSQI score between the WM-EPVS group and the control group, the proportion of poor sleep quality (PSQI score ≥6 points) was higher in the WM-EPVS group than that in the control group (50.6 vs. 35.3%, *p* = 0.031). Spearman's correlation analysis showed that poor sleep quality was positively correlated to WM-EPVS (ρ = 0.154, *p* = 0.044). The binary logistic regression analysis indicated that poor sleep quality was independently related to WM-EPVS (Table 4).

**Table 4:** Results of the binary logistic regression analysis between sleep quality and enlarged perivascular spaces in white matter (WM-EPVS).

**Sleep variables**	**B**	***P***	**Odds ratio**	**95% Confidence intervals**
Poor sleep quality (%)	0.632	0.048	1.882	1.005–3.527
Sleep duration	0.408	0.016	1.504	1.080–2.094
Sleep disturbance	0.814	0.010	2.257	1.220–4.175

*The binary logistic regression analysis was performed adjusting for current smoking use*.

About the relation between sleep components and WM-EPVS, the score of sleep duration [1 (0–2) vs. 1 (0–1.5), *p* = 0.025] and sleep disturbances [1 (0–1) vs. 0 (0–1), *p* = 0.006] were higher in the WM-EPVS group than that in the control group. The results of the binary logistic regression analysis indicated that less sleep duration and sleep disturbance were independent risk factors for WM-EPVS (Table 4).

## Discussion

In this study, we explored the relationship between sleep quality and BG-EPVS and WM-EPVS, respectively, in a lacunar infarction population. We found that poor sleep quality was positively related to BG-EPVS and WM-EPVS. The binary logistic regression analysis showed that poor sleep quality was independently associated with BG-EPVS and WM-EPVS after adjusting for confounders. In addition, we found patients with BG-EPVS had longer sleep latency, less sleep duration, and lower sleep efficiency. Patients with severe WM-EPVS had less sleep duration and more serious sleep disturbances. Binary logistic regression analysis showed that longer sleep latency and less sleep duration were independently related to BG-EPVS, and less sleep duration and more serious sleep disturbances were independently related to WM-EPVS.

Perivascular spaces are thought to serve as a protolymphatic system and play an important role in maintaining neural homeostasis (1). The sulcal CSF is either cleared through the arachnoid granulations or enters the parenchyma via the perivascular spaces, where it combines with interstitial fluid prior to exiting the brain. This perivascular drainage system also allows for the clearance of toxic metabolites within the parenchyma and possibly plays a role in the immunological response of the brain (19–21). EPVSs are thought to be the result of a perivascular blockage, which can be exacerbated by beta-amyloid deposition around cerebral vessels, arteriosclerosis, and decreased arterial pulsatility. In addition, venular amyloid and collagenosis may also contribute to the development of EPVS (22, 23). Although a few EPVS visible on MRI can be normal, the presence of many is not normal and they are associated with some age-related disorders, including cognitive dysfunction, Parkinson's syndrome, WMH, and lacunar infarction (1, 3). It is very important to explore the risk factors for the large amount of EPVS. In this study, EPVS > 10 on one side of the slice containing the maximum amount was defined as the EPVS group. The cutoff >10 EPVS has been used as a high grade in previous studies and showed an excellent intra-rater Cohen k score.

Previously, several studies investigated the relationship between sleep and other MRI markers of cerebral small vessel diseases, such as WMH, lacunar infarction, and deep microbleeds (12, 18). However, to the best of our knowledge, the clinical studies specifically addressing the association between EPVS and sleep quality were scarce. Courtney Berezuk et al. (24) explored the relationship between EPVS in basal ganglia and white matter and sleep by polysomnography among 26 patients with stroke or vascular risk factors. Findings from the study suggested that sleep efficiency was negatively correlated with total EPVS and BG-EPVS, and wake after sleep onset was positively correlated with BG-EPVS. Oscar H. Del Brutto et al. (25) assessed the association between sleep parameters with the PSQI and enlarged BG-PVS in older adults. They found that poor sleep efficiency was independently associated with enlarged BG-PVS, suggesting that sleep may influence structural changes in these fluid-filled cavities. In this study, we analyzed the relationship between sleep quality and BG-EPVS and WM-EPVS, respectively. We found that poor sleep quality was not only independently related to BG-EPVS, but also WM-EPVS. In addition, our results showed that the relation between sleep variables and BG-EPVS was not exactly the same as the relation between sleep variables and WM-EPVS. This might be related to the different pathogenesis of EPVS at the different brain regions. WM-EPVS might reflect cerebral amyloid angiopathy (26), whereas BG-EPVS mainly indicate hypertensive arteriopathy (2), which might be related to the different anatomical structures of EPVS in basal ganglia and white matter. The arteries in the basal ganglia are surrounded by two distinct coats of leptomeninges separated by a perivascular space which is continuous with the perivascular space around arteries in the subarachnoid space, whereas there is only a single periarterial layer of leptomeninges surrounding the arteries in the cerebral cortex and they penetrate into the white matter (27). It was speculated that the anatomical variability may account for differences in clearance efficiency along these drainage pathways. In addition, the influence of age and hypertension on BG-EPVS seems to be stronger than that on WM-EPVS (28). The association between BG-EPVS and WMH also appears to be stronger than that between WM-EPVS and WMH. The exact reason and mechanisms should be further explored in the future.

The potential pathophysiological mechanisms underlying the association between sleep and EPVS are complex and still not completely understood. In an experimental mice model, Xie et al. examined the clearance rates of exogenous and endogenous (beta-amyloid) tracers during awake, sleep, and anesthetized condition (10). They found that the rate of clearance in mice was greatest during sleep. When mice were awake, the tracer influx into the perivascular space decreased compared to natural and anesthesia-induced sleep. The clearance rate of beta-amyloid in the sleeping mice was two times as quick as that in awake mice. These results were explained by a 60% increase in interstitial space volume fraction during sleep, indicating decreased tissue resistance, allowing for greater fluid influx/efflux. This sleep-deprived increase in interstitial space volume may be modulated by changes in sleep-related neurotransmitters, simultaneously modulating sleep onset and metabolite clearance. Therefore, it is reasonable that poor sleep quality contributes to the development of EPVS by influencing the clearance rates of exogenous and endogenous tracers in the brain. This study suggested that poor sleep quality and less sleep duration were independently related to EPVS in both basal ganglia and white matter, which supported the hypothesis. Of course, this should be further demonstrated in the future.

There were some limitations in this study. First, this study was based on patients with lacunar infarction in a small single center, and the cohort may not represent the general population. Second, this was not a prospective cohort study, and the causal relationship between poor sleep quality and EPVS could not be established. Third, sleep quality was assessed by the PSQI. The self-reported sleep variables are less accurate than overnight polysomnography recordings. Despite these limitations, this study investigated the relationship between sleep quality and EPVS in a larger sample compared to the previous study in humans. The PSQI questionnaires have the advantage of being easy to use in clinical practice and reflect the subjective feelings, discomfort, and dissatisfaction with their sleep. We found poor sleep quality was independently associated with EPVS, which would provide some information about the risk factors and pathogenesis for EPVS.

In summary, we found that poor sleep quality was independently related to BG-EPVS and WM-EPVS in patients with lacunar stroke. In addition, we found longer sleep latency and less sleep duration were independently related to BG-EPVS, and less sleep duration and more serious sleep disturbances were independently related to WM-EPVS. The relationship should be further assessed by longitudinal studies in the future.

## Data Availability Statement

The original contributions presented in the study are included in the article/Supplementary Materials, further inquiries can be directed to the corresponding author/s.

## Ethics Statement

The studies involving human participants were reviewed and approved by the Ethics Committee of Beijing Chaoyang Hospital Affiliated to Capital Medical University. The patients/participants provided their written informed consent to participate in this study.

## Author Contributions

WH and SY conceived and designed the experiments. JY and SY participated in the data collection. WQ and LY assessed the imagings. JY participated in the analysis of the data. SY drafted the manuscript. WH and JY revised the manuscript. All authors read and approved the final manuscript to be published.

## Funding

This study was supported by the National Natural Science Foundation of China (Grant No. 81271309).

## Conflict of Interest

The authors declare that the research was conducted in the absence of any commercial or financial relationships that could be construed as a potential conflict of interest. The handling editor declared a shared parent affiliation with several of the authors SY, WQ, LY, and WH at time of review.

## Publisher's Note

All claims expressed in this article are solely those of the authors and do not necessarily represent those of their affiliated organizations, or those of the publisher, the editors and the reviewers. Any product that may be evaluated in this article, or claim that may be made by its manufacturer, is not guaranteed or endorsed by the publisher.
